# The ruminant placental trophoblast binucleate cell: an evolutionary breakthrough

**DOI:** 10.1093/biolre/ioac107

**Published:** 2022-05-19

**Authors:** F B P Wooding

**Affiliations:** Department of Physiology, Development and Neuroscience, The Physiological Laboratory, University of Cambridge, Cambridge, UK

**Keywords:** ruminant placenta, trophoblast binucleate cells, placental evolution, fetomaternal cell, cell fusion

## Abstract

Viviparity and the development of a placenta are two of the major reasons for the success of the mammals in colonizing all habitats, both terrestrial and aquatic. The placenta is an apposition of fetal to maternal tissue which serves two main, but competing functions: to maximize oxygen transfer and the acquisition of nutrients from the mother, but to minimize immunological rejection by the maternal immune system. This has resulted in the evolution of four main types differing in the degree of loss of the maternal uterine epithelial (UE) barrier: epitheliochorial, synepitheliochorial, endotheliochorial, and hemochorial, all providing a successful safe balance between the needs of mother and fetus. Epitheliochorial is the least invasive, a simple apposition and microvillar interdigitation of the apices of uterine epithelium and trophoblast. It is suggested to have evolved as a response to the increase in the size of the animal to provide a sufficiently long gestation to produce a single altricial (run/swim-soon-as-born) neonate as in the Cetartiodactyla. The mother needs to have good control of the fetal demands so the UE barrier is maintained. However, in the synepitheliochorial placenta, characteristic of all ruminants, the fetus has evolved a means of increasing, or at least maintaining, demand without the need for invasion. This has been achieved by the development of the trophoblast binucleate cell which, uniquely, can fuse with a UE cell to form fetomaternal hybrid tissue. This can maintain some maternal barrier function but also deliver fetally synthesized immunomodulatory and metabolic messages to the maternal circulation. This review provides the evidence for this remarkable evolutionary step and also considers an alternative explanation for the formation of the structure of the ruminant placenta.

## Introduction

In this review, individual cells will be identified by the number of nuclei in the cell: for example, Uni, Bi, Tri, or Multinucleate. Other criteria such as large or mature may also be used but not a term such as giant, which is insufficiently informative.

The mid-term ruminant placenta is formed by a species-specific number of placentomes connected by flat interplacentomal areas. The placentomes consist of interdigitated fetal and maternal villi vastly increasing the surface area available for maternofetal transport. The placental uterine epithelium is formed either by uninucleate cuboidal cells occasionally interrupted by fetomaternal trinucleate cells (cow, deer) or by fetomaternal syncytial plaques (ewe, goats), see below for details.

The apposed trophoblast is a sheet of columnar uninucleate cells that also contain 15–20% characteristic ruminant binucleate cells (BNCs).

**Figure 1:**
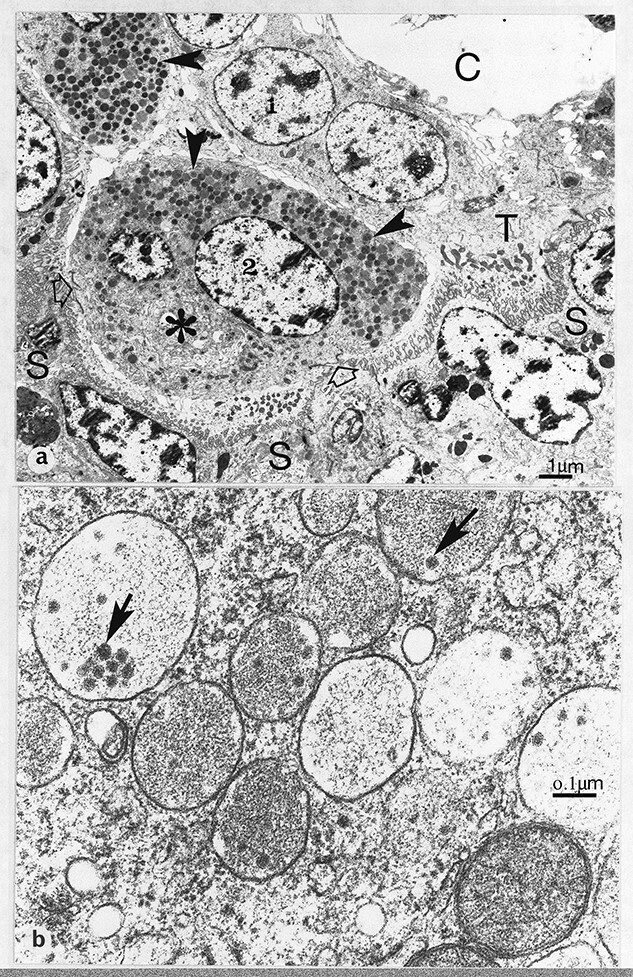
(a) Synepithelio-choriaI placentation. Glutaraldehyde/osmium fixation. Development of fetal binucleate cells (1, young; 2, mature) in the trophectoderm (T) of the definitive placenta of the goat. Note the numerous characteristic granules (arrowheads) and large Golgi body (asterisk) in the mature binucleate cell, which has started to migrate up to the microvillar junction at two points (open arrows). S, fetomaternal syncytial layer; C, fetal connective tissue. 127 dpc, (b) Cow binucleate cell granules containing characteristic rnicrovesicles (arrows). 49 dpc, From [2] Courtesy of Springer Nature.

**Figure 2:**
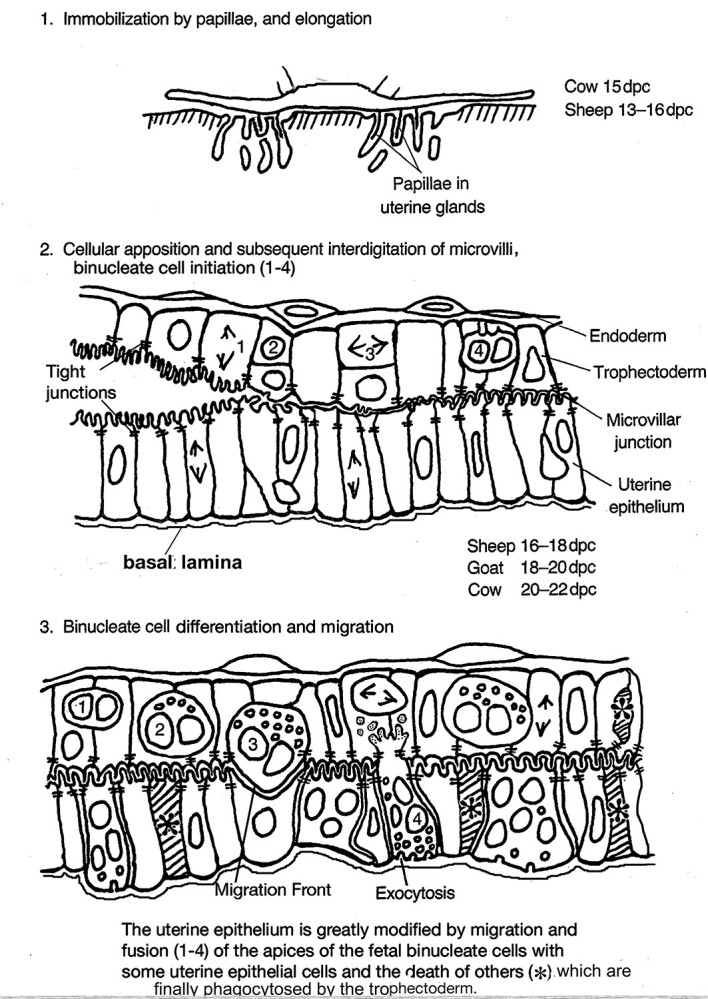
Cellular changes at implantation in the ruminants.. Modified from [2] Courtesy of Springer Nature

**Figure 3:**
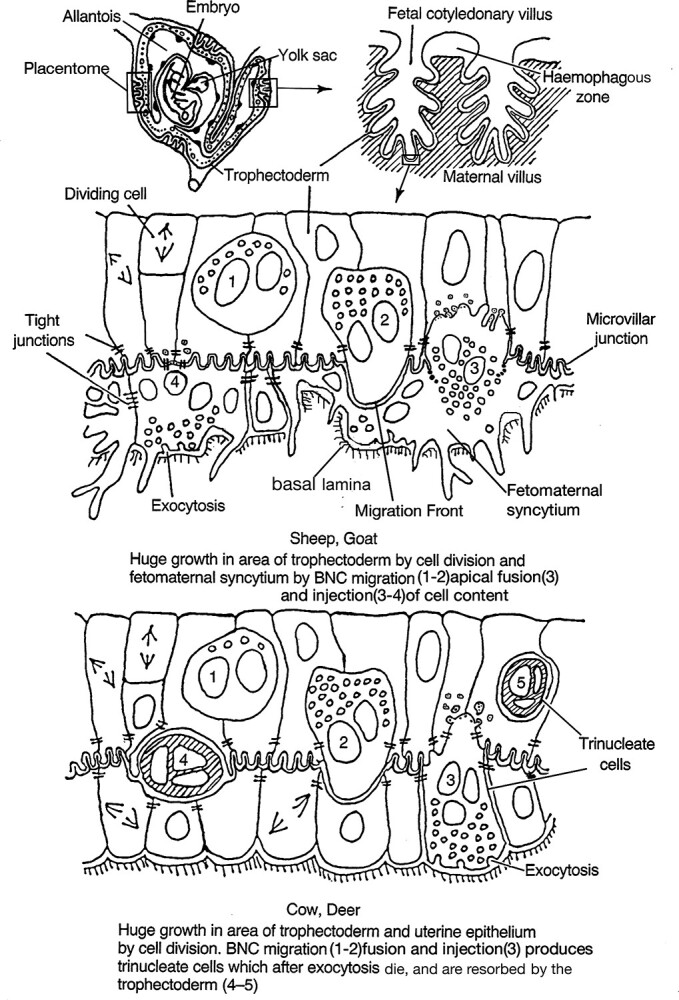
Binucleate cell contribution to the definitive ruminant placenta. Modified from [2] Courtesy of Springer Nature.

These ruminant trophoblast BNCs, when fully differentiated (granulated, Figure 1), are the basis for the formation of the unique synepitheliochorial form of placentation characteristic of the ruminantia [1, 2]. The fully granulated BNC is programmed to migrate through, while maintaining, the trophoblast tight junction (TJ) seal and then fuse with a maternal uterine epithelial (UE) cell or derivative producing fetomaternal hybrid tissue throughout pregnancy (Figures 2 and 3). This allows the delivery of immunologically camouflaged fetal messages in the granules (which contain lactogen hormones and pregnancy-associated glycoproteins (PAGs), exosomes, and other relevant content) throughout gestation to the maternal circulation by exocytosis. These help to maintain the balance between the immunologically foreign fetus and the maternal metabolism.

## Placental types

In evolutionary terms, the synepitheliochorial placenta can be regarded as a development of the epitheliochorial type found in the Perissodactyla, Camelidae, and Suidae. The epitheliochorial placenta is characterized by apposed maternal uterine and fetal trophoblast epithelia both sealed with TJs [2]. These form a considerable barrier to any adverse exchange between mother and immunologically foreign fetus. This potential barrier to oxygen and nutrient transfer is minimized by attenuating the UE/tropho layers specifically between maternal and fetal circulations [2].

The Perissodactyla briefly produce a strictly limited area of trophoblast BNC, which push aside, but do not fuse with, the uterine epithelium as they migrate into the endometrial stroma, forming a discrete mass or “cup” [3]. Here, for a limited period, they secrete only equine chorionic gonadotropin, essential for pregnancy maintenance, before being killed by a maternal immunological response [4].

Camelidae develop large multinucleate individual trophoblast cells [5] which produce only steroids throughout gestation to help to maintain the fetomaternal immunological balance [6].

The Suidae, Hippopotamidae, and Cetacea have epitheliochorial placentas with no reported development of any specifically differentiated trophoblast cells [2].

## Speciation

Using speciation as a criterion for the success of these four types of epitheliochorial placentas after the K/Pg Dinosaur exit, the ruminants are the most successful with 200 or more species in a wide variety of habitats. The Perissodactyla have ~9, the Suidae ~30, the Camelidae ~7, and the Cetacea ~90 [7].

Development of the rumen, a forestomach anaerobic-microbial-fermentation-vat in the gut to utilize the cellulose in a plant fiber rich diet, was obviously an equally important evolutionary development in the speciation of the ruminants [8]. However, Perissodactyla have hind stomach cecal fermentation which can be as efficient as the rumen [9]. Notably, the thousands of Zebra grazing alongside the even greater thousands of ruminant Wildebeest on the African plains is testimony to that. But even when Perissodactyla are competing directly with ruminants this is usually only in a very specialized niche at the top end of the fiber tolerance range, which allows little room for diversification into numerous species outside this niche [9].

The Camelidae [10] and Hippopotamidae [11] also have stomach fermentation but again are limited to very specialized ecologic niches. The Camelidae have also been reported to have the lowest fertility rate compared to other domesticated species [12].

It is of interest that the Cetacea, with as many as 88 species, do show a similar variety of intestinal solutions as do the ruminantia. The killer whales also have a four-chambered stomach [13] but there is no microbial fermentation, they have developed a very muscular forestomach to kill and dismember their largely squid prey, since they cannot chew the soft-bodied food that is swallowed whole. The other main whale group, the Balaenopterids, feeds largely on Krill: minute crustaceans and amphipods. They have evolved a forestomach with a considerable anaerobic- microbial-population producing short-chain fatty acids as in the ruminants [14, 15]. to facilitate digestion. However, all whales show a simple epitheliochorial placenta with no specialized trophoblast cells. Maybe if they had evolved an equivalent strategy to the ruminant trophoblast BNC, their speciation numbers would equal the ruminants.

## Binucleate cell relevance

The BNCs, present in all ruminants, seem to be the particular evolutionary development which, together with their intestinal flexibility, allow the ruminants to be so capable of widespread speciation. Extensive quantitative investigations using light and electron microscopy and immunocytochemistry [2, 18, 19, 22, 23, 24, 29, 31] have established the remarkable uniformity of structure and behavior of BNC in all ruminants so far investigated. The BNCs develop in the ruminant trophectoderm soon after the conceptus anchors itself in the uterus by growing cellular extensions (papillae) down into gland mouths (Figure 2). This promotes close adhesion of trophoblast to UE, initiates the BNC migration, and provides a platform for the advantages the development of the BNC provides.

The first advantage BNC offer is the fact that they appear to be capable of arising from any uninucleate trophoblast cell (UNC, 2N, diploid) by a cell division without cytokinesis which produces an immature BNC presumably with two 2N nuclei. This process occurs throughout pregnancy from implantation to term and results in a constant 15–20% of the trophoblast being BNC at various stages of maturation (Figures 1 and 10) [18]. This process produces a BNC below the trophoblast TJ, without any desmosomal attachments to closely adjacent trophoblast UNC and not in touch with the trophoblast basement lamina [19]. Maturation involves mitotic poyploidization to two larger 4N nuclei [16, 17] coincident with a considerable increase in size with the production of numerous characteristic granules formed from the golgi body (Figures 1 and 10). Occasional rare individual trinucleate cells have been reported but only in the cow [16, 17] and are considered to be formed as a consequence of aberrant mitotic poyploidization processes which normally produce BNC.

**Figure 4:**
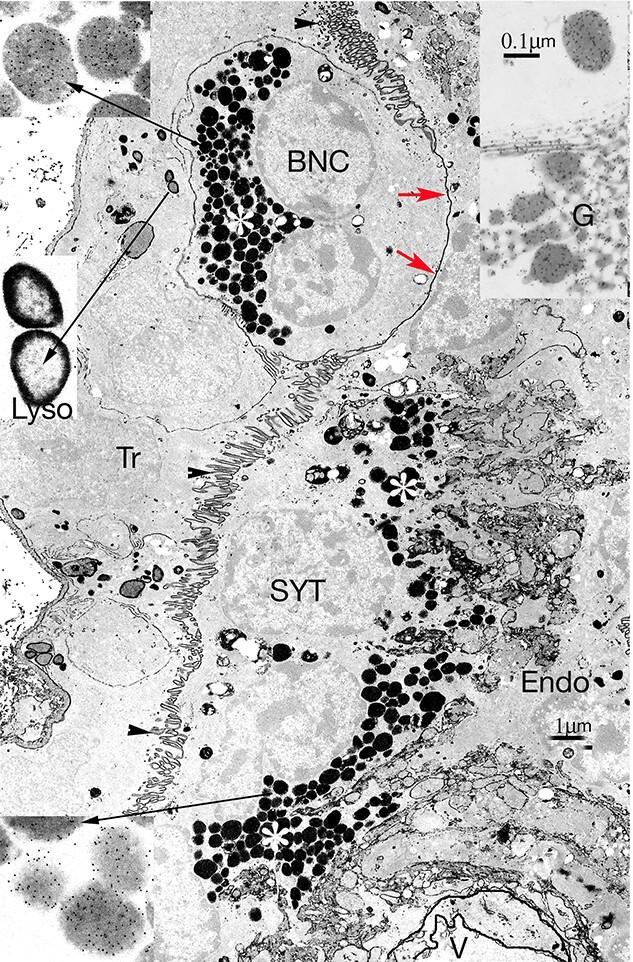
Synepitheliochorial sheep placenta. EM Immunocytochemistry for ovine placental lactogen (OPL) followed by phosphotungstic acid staining. A trophoblast (Tr) binucleate cell (BNC), with a full complement of granules (white asterisk) is migrating across the microvillar junction (arrowheads) by forming a migration front (MF, red arrows). Migration occurs continuously throughout pregnancy. Fusion by vesiculation of a MF has released the characteristic BNC granules from previous migrations (white asterisk) into the fetomaternal syncytium (SYT). These granules will be exocytosed into the maternal endometrium (Endo). V marks a maternal blood vessel. 114 dpc. Higher magnifications at the long arrows indicate that both the Tr and SYT BNC granules show a similar level of OPL labeling, but the lysosomes (Lyso) show no label. Inset on the top right corner is part of a BNC golgi body (G), from a different ovine BNC, also showing OPL label (arrow). The much smaller golgi in the SYT never show label with OPL or PAG antibodies.

**Figure 5:**
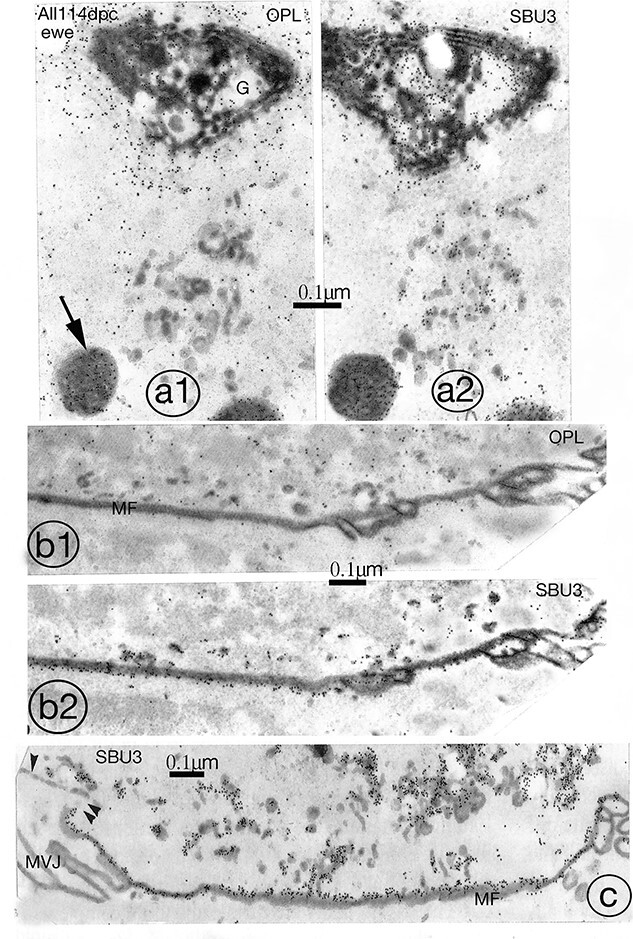
Differential labeling of the sheep binucleate cell migration front (MF) which is the new plasmalemma the migrating BNC forms past the tight junction (Figures 2–4). This new plasmalemma will fuse and break down into vesicles with the apical membrane of the maternofetal SYT to which it is apposed (see Figure 6c, d). The fusion releases the BNC cytoplasmic content into the SYT. Serial sections show that OPL antibody labels (a1, b1) the golgi body (G) and granules (arrow) but not the group of tiny vesicles (v) near the golgi, nor (b1,2), the migration front (MF) whereas SBU3 antibody labels all three (a2, b2, and c) Quantitation of the label [20] shows the specificity of the SBU3 label with the MF at 19 grains per μm and none of the other membranes, including the BNC basolateral plasmalemma (single arrowhead on (c)) and the adjacent microvillar junction (MVJ) on (c) showing levels significantly above background. 114 dpc, From [2] Courtesy of Springer Nature.

A second advantage is the ability of the mature BNC to migrate and fuse with a UE cell. To achieve this, the BNC has co-opt an endogenous retroviral gene, *Syt-Rum-1* [20]. The mature BNC inserts a pseudopodium into the trophoblast apical TJ, maintaining and sharing the junction with the adjacent UNC as it does so (Figures 4, 8, and 9). The pseudopodium increases in size and penetration and, at implantation, flattens the apposed uterine microvilli on a single uterine cell. EM immunocytochemistry shows that this pseudopodial membrane or “migration front” is formed from tiny membrane vesicles produced close to the golgi in the ewe (Figure 5) [21].

**Figure 6:**
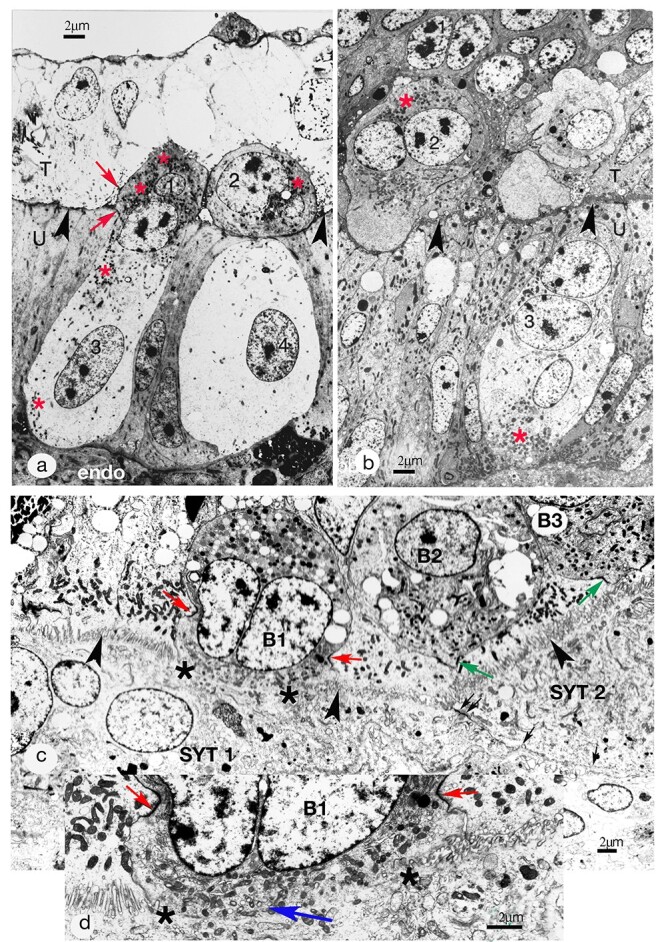
(a) Synepitheliochorial placentation. Implantation (16 dpc) in the sheep. Fusion of two fetal binucleate cells (1 and 2), each with a uterine epithelial (UE) cell (3 and 4). The fused fetomaternal cell shares tight junctions (TJs) with the UE and the trophoblast (red arrows). Cells 2 and 4 were continuous on a different plane of section [21]. The material is non-osmicated and stained with phosphotungstic acid to emphasize the binucleate cell granules (red asterisks), nuclei, and microvillar junction (arrowheads), the cytoplasm appears empty. However, after osmium, conventional uranyl acetate and lead section staining produces from the same material micrographs equivalent to those in Figures 1 and 6b–d. T, fetal trophoblast,; U, uterine epithelium; Endo, endometrium. 16 dpc, Modified from [2] Courtesy of Springer Nature. (b) Synepitheliochorial placentation. Implantation (20 dpc) in the cow. Glutaraldehyde and osmium fixation. Binucleate cells, young (1) and mature (2) with characteristic apical granules (red asterisk). BNC 2 is migrating up to the microvillar junction (arrowheads) between trophectoderm (T) and uterine epithelium (U). The uterine epithelium includes a trinucleate cell (3) with basal granules (red asterisk) and two round nuclei very similar to those in the binucleate cell. This trinucleate is probably a fetomatemal hybrid cell produced by fetal binucleate cell fusion with a uterine epithelial cell (see [26]). 20 dpc. Modified from [2] Courtesy of Springer Nature. (c) Synepitheliochorial placenta, Implantation in the 19 dpc goat. Glutaraldehyde and osmium fixation. The uterine epithelium has already been modified to fetomaternal (SYTs 1 and 2) and trophoblast BNC B1 has just fused into the syncytial plaque 1. What was the migration front of BNC B1 has vesiculated (between the asterisks) and interrupts the MVJ (indicated by arrowheads). The trophoblast TJ through which B1 is migrating is indicated by the red arrows. Trophoblast BNCs B2 and B3 are also ready to migrate and have pushed pseudopodia into the trophoblast TJs (green arrows). There is also a TJ (double arrow) sealing the apposed plasmalemmas (arrows) of the two syncytial plaques. (d) Inset: a higher magnification of the fusion area (between asterisks) of BNC B1. The TJ through which BNC 1 is migrating is more obvious (red arrows) and there is a collection of vesicles (blue arrow) which may be remnants of the migration front.

The resultant flat apposition between the uterine and BNC pseudopodial membrane front then breaks down into vesicles presumably under the influence of the *Syt-Rum-1* gene and the content of the BNC is expelled into the UE cell (Figures 2, 3, and 6). This produces a fetomaternal trinucleate cell still presenting only maternal plasmalemmal antibodies to the maternal immunological defenses, a third advantage of the system. Quantitative histological investigations of serial sections at this early implantation stage in sheep and goat found only uni- and BNCs in the trophoblast epithelium but uni, tri-, and pentanucleate cells in the uterine epithelium, the last as a result of a further BNC migration and fusion. No evidence for any uterine BNCs was found indicating that no UE cell fusion occurs [22].

This leads to a fourth advantage as these processes deliver the fetally synthesized BNC granules to the base of the fetomaternal tissue where they exocytose their content to the maternal tissue [22, 23, 24] (Figures 2, 3, and 9). Immunocytochemical investigation shows their content includes placental lactogens [25] (Figure 4) prolactin related proteins [26], PAGs (Figure 4, SBU-3) [27], and exosome-sized microvesicles [53, 55] (Figure 9) all potentially capable of modifying maternal metabolism and immunological defenses.

This content also has been shown capable of modification during pregnancy [27, 53] and this variety and flexibility forms a fifth advantage of the ruminant BNC system.

## Implantation and placentomal development

**Figure 7:**
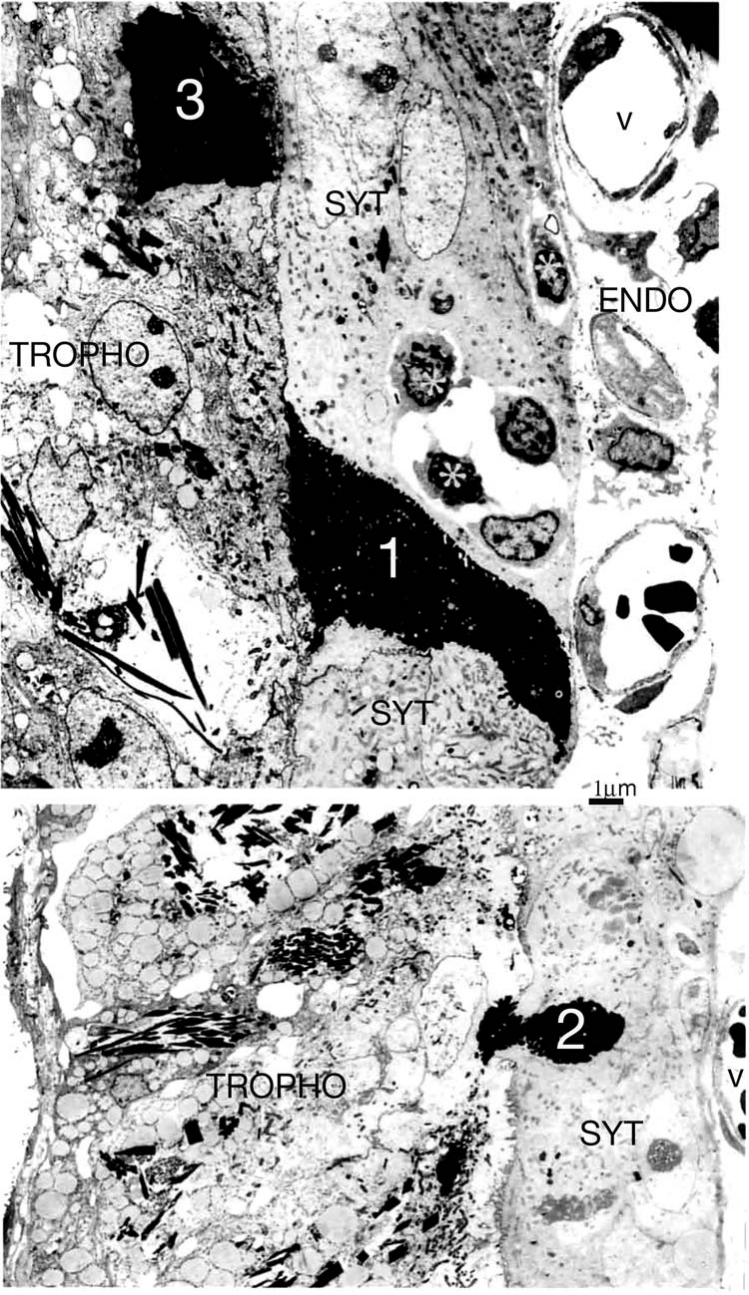
Implantation in the 22 dpc goat. As the BNCs migrate and fuse the UE is transformed into a syncytium (SYT). Other UE cells die, become dense and shrunken (1), are extruded into (2) and phagocytosed (3) by the trophoblast. There are several intraepithelial lymphocytes (asterisks), apparently associated with the syncytium.

The implantation process, which is initially restricted to specific flat areas, the uterine caruncles (which are devoid of endometrial glands), continues with further BNC migration and fusion. This produces syncytial plaques in place of the UE cells which are either incorporated into the syncytium or eliminated by death and phagocytosis by the trophoblast cells (Figure 7). In the ewe, serial section counting indicates that the syncytial plaques are a fairly uniform size, each containing ~25 nuclei [22]. These processes have been clearly documented in ewe [22], goat [23, 24] (Figure 7), cow [28] and deer [29].

The next requirement is an increase in the surface area of the placental membranes to allow a steady increase in maternofetal food transport as the fetus grows. This is accomplished by a mutual growth of caruncular trophoblast and the uterine epithelium or derivative to form the placentomes which consist of enmeshed fetal and maternal placentomal villi (Figure 3).

In the case of the ewe and goat, this growth is based on continual trophoblast division and growth, producing BNCs that migrate and fuse to form the maternofetal syncytial plaques (Figure 9). No one has ever reported nuclear division in the plaques; they only form by BNC migration. Injection of radioactive thymidine into the ewe fetus in vivo and subsequent sampling of the placenta at set time periods allows this growth process to be followed exactly using autoradiography [30]. The label is found initially in the trophoblast UNC, then in the BNC, and finally in the syncytial plaque nuclei in the ewe and goat. This confirms the BNC fusion and migration hypothesis in a more dynamic way than the numerous but static electron micrographs on which it is based.

It is not yet clear what advantages the syncytial plaque system provides. A continuous syncytium as found in the endothelial and hemochorial placentas is a more effective fetal barrier to maternal cellular translocation and is also active in synthesizing fetal responses to maternal immunological attack. There is no evidence that the ruminant syncytial plaques synthesize anything specific, but their formation results from continuous BNC migration, fusion, and delivery of granules containing fetally produced mediators of maternal metabolism. They are fetomaternal tissue and may provide a maternal immunological buffer zone. They may contain only one maternal nucleus to the 22–25 fetal nuclei in each plaque if they are formed by sequential BNC fusion into one original uterine trinucleate cell (TNC). Alternatively, if lateral fusion between uterine TNC is possible, then plaques could contain one maternal nucleus to each two BNC nuclei. The exact nature of each plaque awaits future research.

**Figure 8:**
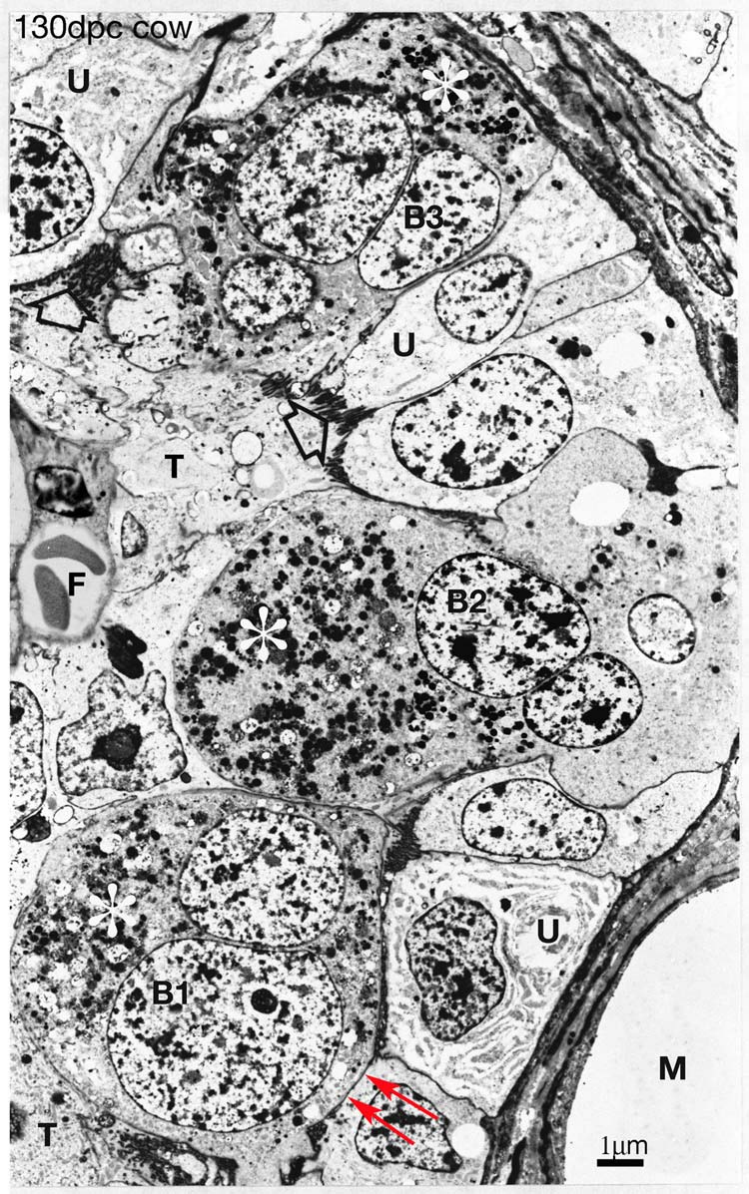
Synepitheliochorial cow placenta 130 dpc. PTA staining. Throughout pregnancy mature fully granulated (white asterisks) binucleate cells (B1, which shows the start of an migration front, red arrows) migrate from the fetal trophectoderm (T) across the microvillar junction (open arrow) to fuse with uterine epithelial cells (U) producing trinucleate cells (B2). These release their granules (B3, asterisk) close to the maternal blood vessels (m), die, and are resorbed by the trophectoderm (Figure 3) [29] F fetal blood vessel. 130 dpc, Modified from [2] Courtesy of Springer Nature

The cow and deer use a different strategy for placental growth but still retain the BNC migration and fusion involvement (Figure 3). The syncytium formed at implantation is rapidly replaced by residual UE cell division and growth plus syncytial death and phagocytosis by the UNC trophoblast. This produces two apposed UNC epithelia, cuboidal uterine and trophoblast, sharing a microvillar interdigitation at their apices. This is the basis for all the considerable villus growth and development of the placentome. The trophoblast maintains the 15–20% population of BNC, which migrate and fuse throughout pregnancy forming fetomaternal trinucleate cells (Figure 8). These exocytose their granule content of fetal mediators to the maternal side, die, and are resorbed by the UNC trophoblast [31]. The intact maternal uterine epithelium would seem a better immunological buffer zone than the syncytial plaques and the BNC migration and fusion system still allows continual delivery of fetal mediators. All of the cervids and most of the Bovids which have been investigated show the cow and deer strategy [32], so this may be the more versatile of the two systems.

## Establishment of BNC behavior

In 1906, Assheton clearly identified BNC on the light microscope (LM) and suggested they might play a role in forming the uterine epithelium syncytium in the Ewe [54]. Later workers [33–37] established the presence and equivalent structure of BNC in the trophoblast in several ruminants (sheep, cows, and deer) but published no LM or EM evidence of the involvement of BNC in syncytium formation. EM work in 1981 established the fact of BNC migration [37] and the delivery of the characteristic BNC granules, shown by immunocytochemistry to contain placental lactogens (Figure 4), to the maternal side of the placenta [38]. This correlated with the demonstration of placental lactogen in the maternal circulation.

**Figure 9:**
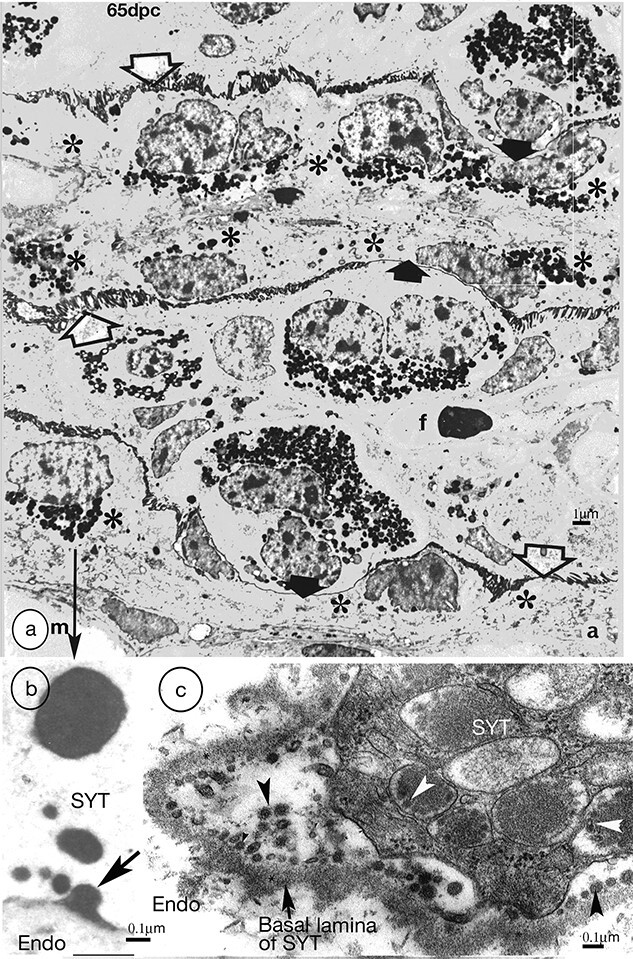
(a) Synepitheliochorial sheep placenta 65 dpc. PTA staining. Binucleate cells migrate (solid arrows show migration fronts) across the microvillar junction (open arrows) throughout pregnancy delivering their granules to the base of the fetomaternal syncytium and forming syncytial plaques (asterisks). m, maternal, f, fetal, blood vessels. Modified from [2] Courtesy of Springer Nature. (b) Higher magnification from (a) An example of exocytosis (arrow) of (originally) BNC granules into the endometrium. SYT, syncytium. Endo endometrium. (c) Goat placenta. Glutaraldehyde and osmium fixation preserves the BNC granule microvesicles/exosomes (arrowheads) and makes recent exocytosis events immediately recognizable.

Introduction of the PhosphoTungstic Acid (PTA) stain on non-osmicated, deresinated Araldite EM sections [39] picked out only the nuclei, the microvillar junction (MVJ) between uterine epithelium and trophoblast and the BNC granules. This allowed the clear demonstration of BNC cell fusion with a uterine cell or derivative in the ewe [22], cow [28] and goat [23, 24] (Figures 6, 8, and 9). Recent LM immunocytochemical work has shown that BNC migration and fusion to form a fetomaternal TNC throughout pregnancy is common to all of a wide variety of ruminant species showing the cow and deer pattern [32]. With the ewe and goat unequivocal EM examples of fusion with the syncytial plaques can readily be found [22–24] (Figure 6c, d) Evidence that the mature BNC maintains the trophoblast TJ barrier as it migrates through it has been provided by Freeze fracture micrographs [40].

Subsequent quantitative studies of serial sections and autoradiographic tracing of BNC nuclear movements have confirmed the basic hypothesis diagrammed in Figures 2 and 3. Isolation of purified populations of ewe and goat BNC have established their potential for producing lactogens, PAGs, and steroids [41]. Lectin histochemistry of tissue BNC glycans allows recognition of specific complex carbohydrate side chains which have been shown to be present on PAGs and prolactin-related protein (but not Lactogens). Such side chains are identified on BNC granules in all of the species so far examined in this way, for example, Tragulus, Cow, Deer, Goat, Springbok, and Impala [42].

Some species do show individual extras in the BNC granules, for example, c-type natriuretic peptide in ewe [43] and glucose transporter-1 in Tragulus [44]. In Giraffe, as expected, all BNCs contain lectin binding granules throughout the fetal villus, but PAGs are only expressed in BNC at the tips and lactogen proteins are expressed only in the basal trophoblast BNC. Since the Giraffe BNCs show the normal migration and fusion process [32], this is another example of the flexibility of the BNC system [45].

## Control of BNC production

What controls the BNC production, maturation, migration, and heterologous fusion behavior is not understood. Many fetal interventions such as adrenalectomy, stalk section, hypophysectomy, or injection of mouse epidermal growth factor (EGF) have little or no effect on BNC formation, maturation or migration [46].

Monolayers of Trophoblast cells grown in vitro do produce occasional BNC, but how equivalent these are to the in vivo mature BNC, which are notably uniform in EM and LM structure in all species so far examined, has not yet been established [47]. This seems a potentially important area for research into control of BNC production and maturation.

## BNC fusion and *syncytin* genes

This review has been designed to show the importance of BNC fusion for successful ruminant implantation and placental growth. Recent work on the endogenous retroviral origin of the *syncytin* genes has provided further support for this. Over many millions of years of evolution, many endogenous retroviral (ERV) genes have been incorporated into the DNA of mammals. A few have retained an important viral function after incorporation into the host genome. ERVenv is one which is expressed in human and mouse placenta and is involved in the placental cell fusion processes forming the syncytial layers which serve as the immunological and functional barrier between mother and fetus [48]. A gene of similar origin is expressed in the developing ewe trophoblast from day 12 of pregnancy. Blocking the function of this gene with morpholino antisense oligonucleotides injected into the uterus on day 8 produces considerably reduced conceptus size with no significant BNC at day 16. These pregnancies fail by day 20 [49]. More specific investigations [20] have identified *syncytin* genes of retroviral origin coopted for a role in placentation present in both ovine and bovine genomes and referred to as Syt-Rum-1. In the bovine only, a second phylogenetically unrelated *syncytin* gene, BERV K-1 (Fematrin1), has been found [56]. Both gene expressions are placenta specific. In situ hybridization studies using labeled riboprobes have clearly identified the BNC as the only cell type expressing Syt-Rum-1 or BERV K1. Neither the trophoblast UNC nor the uterine epithelium or derivative expresses the genes. The results of these studies [20, 56] suggest that the co-option of the *syncytin* genes into the ruminant trophoblast could have provided a major driving force for the evolution of the unique BNCs that are the basis for the formation of the synepitheliochorial placenta.

## Alternative theory of ruminant placental development

The BNC migration and fusion hypothesis has recently been seriously questioned based on the results from an immunofluorescent study of implantation in the ewe by Seo et al. [50] They suggest that the syncytial plaques in ewe implantation originate from trophoblast giant cells (TGCs) with three or four nuclei, which form by trophoblast UNC fusion. These TGCs migrate into and then eliminate the UE. The TGCs then fuse to form the syncytial plaques in place of the UE. Figure 11 is a diagram illustrating this “TGC hypothesis” from the Seo et al. paper (with permission from Prof G Johnson).

The authors dismiss all the EM evidence for the migration and fusion of BNC hypothesis because it was “without the benefit of molecular markers for BNCs and UE cells.” This ignores the LM and EM immunocytochemistry of the BNC granules [25–27] and the emphasis on defining the TJs of the trophoblast and uterine epithelium as markers of the fusion process. It also ignores the LM autoradiography study defining the BNC migration [30], the freeze etch study [40] confirming the BNC migration through the trophoblast TJ and the EM immunocytochemistry characterizing the “migration front” of the BNC (Figure 5) [21].

Seo et al. interpret their elegant immunofluorescent study without allowing for the problem of its inherent lack of resolution. For example, the position of the MVJ between trophoblast and UE cells or derivative has to be guessed (dotted lines in Figure 12A) rather than clearly defined as in our EM PTA studies (Figure 12B and C).

This is critical in the Seo et al. Figure 1A (reproduced in Figure 11A, with permission) which they interpret as showing tri and quadrinucleated cells “in the trophoblast.” This image can be better interpreted using PTA stained electron micrographs as BNC cells fusing into syncytial plaques, similar to the EM PTA images in Figure 12B and C, in which the MVJ has clearly reformed behind the fused BNC. At the start of BNC–UE fusions the bulk of the BNC will always be in the trophoblast layer. The incorporation of the fused fetomaternal cell into the UE cell layer involves considerable reorganization of that layer including phagocytosis of the original BNC basolateral membrane by the trophoblast and reformation of the MVJ.

Considering the five bullet points stated in the abstract of the Seo et al. paper:

*Bullet Point 1*. A basic problem with the TGC hypothesis is the lack of any evidence for TGC formation. Occasional individual trinucleate cells have been reported but only in the cow, and they are considered to be formed as a consequence of aberrant mitotic poyploidization processes which normally produce BNC [16, 17].

**Figure 10:**
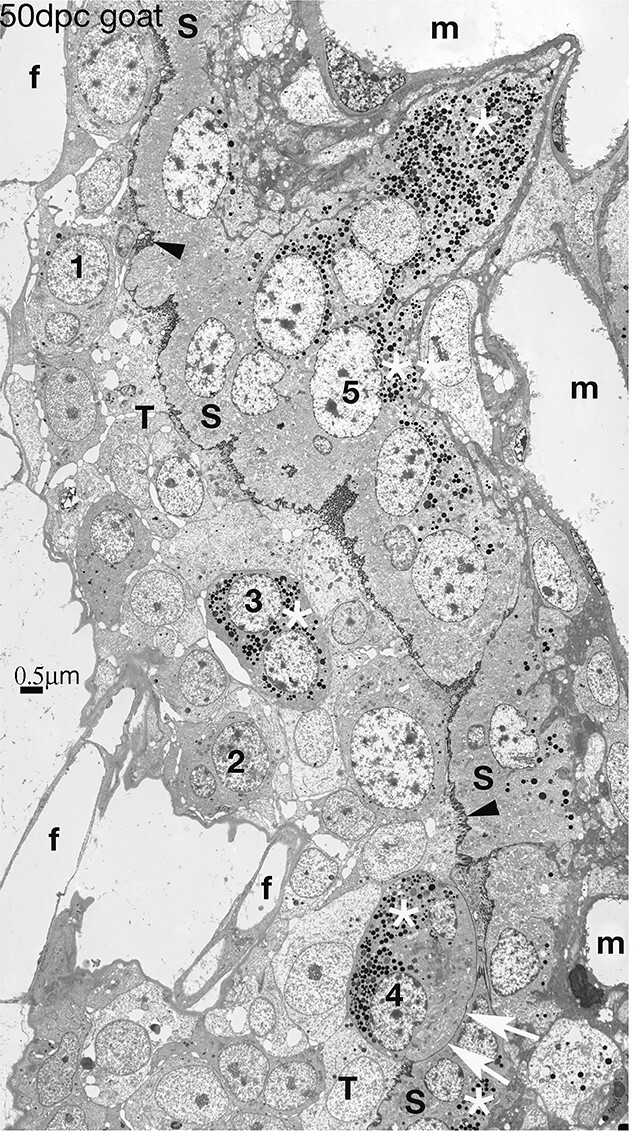
Synepitheliochorial placenta in the goat. Numerous BNC are present in the trophectoderm, a sequence of development 1–4 is shown, with a fully granulated (white asterisk) BNC4 showing an Migration Front (white arrows) which will vesiculate and release the content of BNC4 including its granules (white asterisk) to form part of the syncytium (S). This contains numerous granules (white asterisks), all derived from previous BNC fusions, and many nuclei (e.g. 5) most of which are from BNC. 50 dpc. Modified from [2] Courtesy of Springer Nature

EM studies have clearly shown that the BNCs differentiate within the trophoblast and below the trophoblast TJ and only migrate and fuse when fully granulated and mature (Figures 4, 8–10) [37]. The presence of trophoblast BNC has been clearly established in numerous LM and EM studies since Assheton in 1906 [54, 33–38]. No paper known to the author has established any evidence for the presence of tri- or quadrinucleated cells (TGC) in the ewe or any other ruminant trophoblast epithelium.

**Figure 11:**
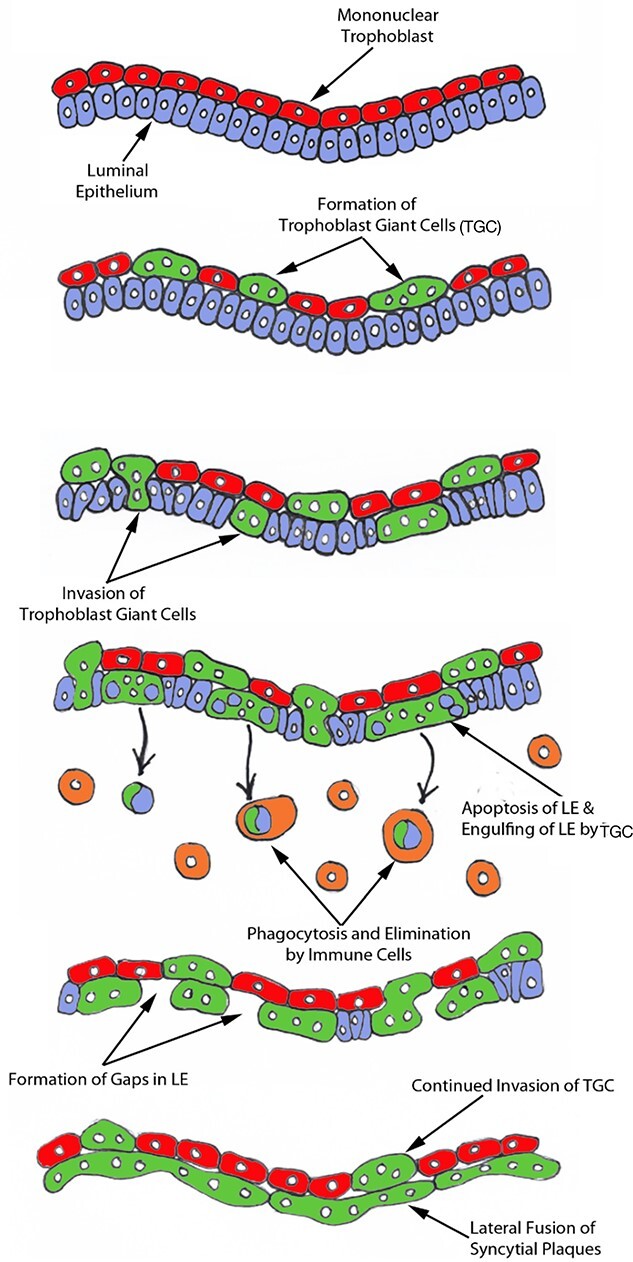
Seo et al. “working hypothesis for the syncytialization of the sheep placenta” With permission from Prof G Johnson.

**Figure 12:**
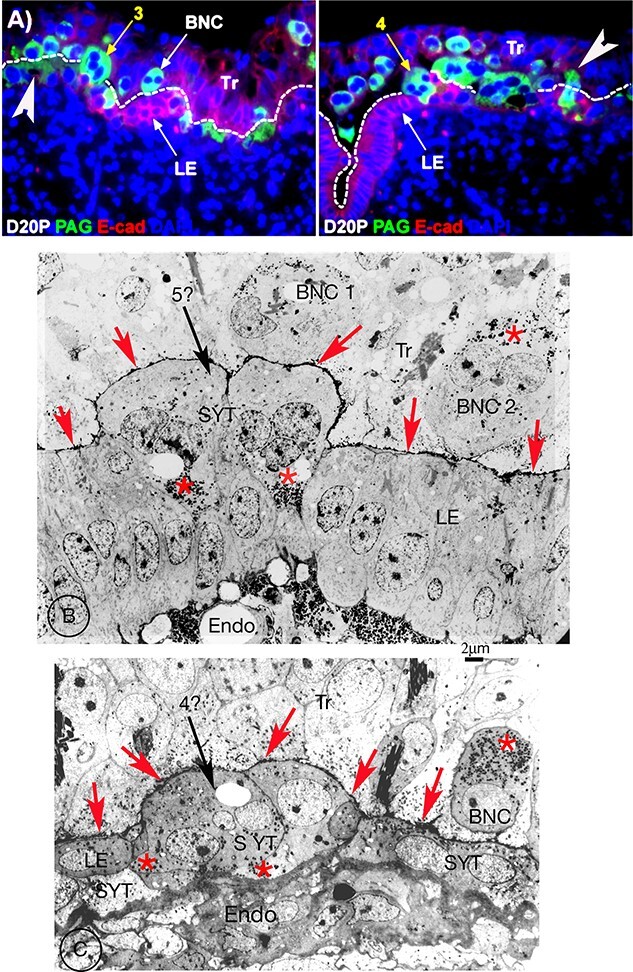
(A) Fluorescent images from the Seo et al. paper, (with permission from Prof G Johnson). Width of each image is 220 μm) day 20 sheep. Immunostained in green with Pregnancy Associated Glycoprotein (PAG) antibody, and in red with E-cadherin. The PAG stains the whole cytoplasm of any “TGC” not just the granules. The guesstimated MVJ between UE and trophoblast is indicated with a dotted line. In two examples (white arrows added by the current author) this MVJ crosses the center of “TGCs”. The cytoplasm of the “TGC” indicated by arrow 3, which does interrupt the MVJ, does not extend to the basement lamina of the UE. The cadherin stain of the UE shows below it. The “TGC” indicated by arrow 4 does extend to the UE basement lamina. (B, C) are both EM images from a 20 dpc sheep, stained with PTA, with BNC granules (red asterisks) apical in trophoblast BNC and basal in the syncytium. The MVJ is clearly marked (red arrowheads) and the endometrium (Endo) obvious. I consider that the arrow 5? Cell in (B) is equivalent to the arrow 3 cell in (A) and the arrow 4? cell in (C) equivalent to arrow 4 cell in (A). All arrowed cells are produced by sequential fusion of BNC into UE cells or the syncytium. In my opinion, the cells indicated by the arrows 3 and 4 in (A) are on the uterine side of the MVJ and not in the trophoblast. Tr, trophoblast; LE, uterine (luminal) epithelium; Endo endometrium; MVJ, microvillar junction; SYT, syncytium.

Are the TGCs formed below the trophoblast TJ? When do they accumulate granules? Do they pass through while maintaining the trophoblast and UE TJs? None of these questions can be answered conclusively with the resolution of LM fluorescent studies.

These considerations emphasize the inaccuracy of the claim for the normal presence of TGC in the trophoblast.

*Bullet point 2.* Death of UE cells during syncytial plaque formation has also clearly been shown by EM studies, but resolved by EM evidence of trophoblast phagocytic uptake of the residues [22, 23, 24, 26, 27] (Figure 7).

*Bullet point 3.* The immunofluorescent resolution is insufficient to establish any clear evidence for TGC engulfment of apoptopic UE residues. A few gaps between forming syncytial plaques have been shown to be normal at ovine implantation [22] However, the UE basal lamina (the more usually used term “basement membrane” is misleading, there is no membrane structure) always remains continuous without gaps which are a reflection of the considerable cellular alterations needed to replace the original UE with syncytial plaques.

*Bullet point 4.* Syncytial plaque fusion is possible, but the plaques do not show the *Syt-Rum-1* gene, which as detailed above, is restricted to BNC [20].

*Bullet point 5.* None of the previous LM and EM studies of ruminant implantation have shown any cells crossing the basal lamina of the UE, nor any accumulation of leucocytes below this layer under normal circumstances. The concept of the TGC delivering UE apoptopic residues to subepithelial leucocytes seems very unlikely especially since the only published example [51] of such an accumulation of leucocytes was found in a study of ewe placentas at 22 days of pregnancy after ovariectomy on day 20.

There is no published evidence for the significant synthesis of immunomodulators by the plaques, they appear to act as platforms for delivery of the content of BNC granules to the maternal circulation. In that respect, they are not at all analogous to the human syncytiotrophoblast which is very active in synthesizing such modulators [52].

## Conclusion

The wealth of detail of ruminant placental development illustrated diagrammatically in Figures 2 and 3 is amply justified by the LM and EM studies cited and reinforced by the figures presented here. All of the LM and EM evidence both characterizes the individual cells involved and suggests how they behave *in vivo*. This supports the BNC migration and fusion hypothesis.

On the other hand, the TGC hypothesis as illustrated in Figure 11 lacks any detail. It does not show, for example, how and where the TGC form, is it below the TJ, or at the MVJ? Why have none of the many previous investigators found any evidence of tri or quadrinucleate trophoblast cells? How are the trophoblast and uterine TJs negotiated by the TGC––does the TGC hypothesis suggest the presence of “loose” TJs at these sites? Do the TGC that “deliver” the apoptopic UE residues to the leucocytes rejoin the forming syncytial plaques replacing the UE?

On balance, it seems that the TGC hypothesis must await further specific results before any of its predictions can be seriously considered.

## Conflict of interest

There is no potential conflict of interest with any public or commercial organizations.

## Funding

This research did not receive any specific grant from any funding agency in the public, commercial, or not-for-profit sector.
